# Antisclerosin for Administration of Blood Salts

**Published:** 1907-05

**Authors:** 


### Antisclerosin for Administration of Blood Salts.

Privy Councillor Senator says in the *Russiche med. Rundschau,* January, 1907, that antisclerosin is the simplest and most convenient method of administering the blood salts, the use of which is based on the work of Dennstedt-Rumph and Ludwig Weil. The antisclerosin tablets contain chiefly sodium chloride, as also sodium sulphate, sodium phosphate, sodium carbonate, magnesium phosphate and calcium glycerophosphate,—all in about the proportion in which they are normally present in the blood. The initial dose is two tablets daily and is gradually increased to six daily. If the dosage is slowly increased, the antisclerosin is well borne. Though the causal relation of salt impoverishment of the blood to arteriosclerosis is not yet wholly explained, salt impoverishment is certainly an abnormal condition. The careful employment of the blood salts can only be of benefit.

---

Aspiration in Otitis Media Acuta.—Percy R. Wood (*Medical Record,* April 13, 1907,) advocates aspiration of the drum head in acute otitis media to secure drainage and control the infection. The disease is generally the result of infection in the acute infectious diseases, or arising from the pressure of adenoid growth in the nasopharynx, which always foster the retention of bacteria near the mouth of the Eustachian tube. When drainage is not secured, chronic disease of the middle ear and mastoid generally result, brought about by the retention of the secretions of the acute process. Early paracentesis, after applying a mixture of menthol, cocaine, and carbolic acid, is painless, and the wound heals in a few days. By means of a middle ear aspirator, described by the author, quantities of pathological secretions may be removed. Warm camphorated vapor is sent through the Eustachian tube, the meatus dusted with boracic acid, and packed with iodoform gause, and the pharynx painted with 2 per cent. nitrate of silver solution. Dressings should be replaced daily.

